# Associations of neuroimaging markers with depressive symptoms over time in middle-aged and elderly persons

**DOI:** 10.1017/S003329172200112X

**Published:** 2023-07

**Authors:** Fatih Özel, Saima Hilal, Maud de Feijter, Isabelle van der Velpen, Nese Direk, M. Arfan Ikram, Meike W. Vernooij, Annemarie I. Luik

**Affiliations:** 1 Department of Organismal Biology, Uppsala University, Uppsala, Sweden; 2 Saw Swee Hock School of Public Health, National University of Singapore and National University Health System, Singapore; 3 Department of Epidemiology, Erasmus University Medical Center, Rotterdam, The Netherlands; 4 Department of Radiology and Nuclear Medicine, Erasmus University Medical Center, Rotterdam, The Netherlands; 5 Istanbul Faculty of Medicine, Department of Psychiatry, Istanbul University, Istanbul, Turkey

**Keywords:** cerebrovascular disease, depression, neuroimaging, white matter

## Abstract

**Background:**

Cerebrovascular disease is regarded as a potential cause of late-life depression. Yet, evidence for associations of neuroimaging markers of vascular brain disease with depressive symptoms is inconclusive. We examined the associations of neuroimaging markers and depressive symptoms in a large population-based study of middle-aged and elderly persons over time.

**Methods:**

A total of 4943 participants (mean age = 64.6 ± 11.1 years, 55.7% women) from the Rotterdam Study were included. At baseline, total brain volume, gray matter volume, white matter volume, white matter hyperintensities volume, cortical infarcts, lacunar infarcts, microbleeds, white matter fractional anisotropy, and mean diffusivity (MD) were measured with a brain MRI (1.5T). Depressive symptoms were assessed twice with the Center for Epidemiologic Studies Depression scale (median follow-up time: 5.5 years, IQR = 0.9). To assess temporal associations of neuroimaging markers and depressive symptoms, linear mixed models were used.

**Results:**

A smaller total brain volume (*β* = −0.107, 95% CI −0.192 to −0.022), larger white matter hyperintensities volume (*β* = 0.047, 95% CI 0.010–0.084), presence of cortical infarcts (*β* = 0.194, 95% CI 0.047–0.341), and higher MD levels (*β* = 0.060, 95% CI 0.022–0.098) were cross-sectionally associated with more depressive symptoms. Longitudinal analyses showed that small total brain volume (*β* = −0.091, 95% CI −0.167 to −0.015) and presence of cortical infarcts (*β* = 0.168, 95% CI 0.022–0.314) were associated with increasing depressive symptoms over time. After stratification on age, effect sizes were more pronounced at older ages.

**Conclusions:**

Neuroimaging markers of white matter microstructural damage were associated with depressive symptoms longitudinally in this study of middle-aged and elderly persons. These associations were more pronounced at older ages, providing evidence for the role of white matter structure in late-life depressive symptomatology.

## Introduction

Nearly 20% of the population is estimated to experience at least one major depressive disorder episode during their life (de Graaf, Ten Have, van Gool, & van Dorsselaer, 2012; Kessler & Bromet, 2013). Across the lifespan, there are two peaks in the incidence of depression; the first peak is around the age of 20 and the second peak is starting around the age of 60 (Pedersen et al., 2014). The natural course of depression worsens with increasing age (Mitchell & Subramaniam, 2005; Schaakxs et al., 2018); those with late-life depression are thought to have more pronounced cognitive deficits and poor treatment response, making it crucial to improve our understanding of late-life depression.

One factor thought to play a role in late-life depression is cerebrovascular disease, which is common at old age and often comorbid with depression. Vascular lesions may cause widespread changes in the brain and potentially cause depression. This has led to the generation of ‘the vascular depression hypothesis’ (Aizenstein et al., 2016; Alexopoulos et al., 1997). The hypothesis postulates that cerebrovascular disease may predispose, precipitate, or perpetuate some geriatric depressive symptoms (Alexopoulos et al., 1997), emphasizing its role in the etiology of late-life depression (Alexopoulos, 2019; Taylor, Aizenstein, & Alexopoulos, 2013).

The vascular depression hypothesis has been supported by a range of neuroimaging findings. First, cardiovascular risk factors have been demonstrated to be associated with brain atrophy and structural changes (Almeida et al., 2008; Enzinger et al., 2005; Gu et al., 2019), which have been thought to contribute to late-life depression. Both brain volume and cortical atrophy have been related to baseline and incident depressive symptoms and disorders (Geerlings et al., 2012; Geerlings & Gerritsen, 2017; Grool et al., 2012; Qiu et al., 2017; Tudorascu et al., 2014; van Sloten et al., 2015). Additionally, gray matter volume has been suggested to be linked to depressive symptoms (Du et al., 2014). However, results are inconclusive as no associations have also been suggested in other work (Ikram et al., 2010). Second, cerebral small vessel disease (CSVD) has been proposed as a risk factor for depression, yet the evidence for an association of markers of CSVD with depression is also heterogeneous (Rensma, van Sloten, Launer, & Stehouwer, 2018). White matter hyperintensities (WMH) have been suggested to be fundamental to vascular depression (van Agtmaal, Houben, Pouwer, Stehouwer, & Schram, 2017), even though some studies could not find an association (Dotson, Zonderman, Kraut, & Resnick, 2013; Versluis et al., 2006). There is also accumulating evidence that lacunar infarcts (Direk et al., 2016; Grool et al., 2013; Pavlovic et al., 2016; van Sloten et al., 2015) and microbleeds (Wang, Liu, Ye, & Yan, 2018) might play a role in late-life depression. Third, markers of microstructural integrity have also been investigated, since cardiovascular risk factors are known to have detrimental effects on white matter (Hannawi et al., 2018; Tamura & Araki, 2015). Indeed, disruptions of white matter tracts seem to contribute to depressive symptomatology late in life. Lower fractional anisotropy (FA) and higher mean diffusivity (MD), positing damage of white matter microstructure, were manifested in elderly depressed individuals (Brookes, Herbert, Lawrence, Morris, & Markus, 2014; Reppermund et al., 2014; Shen et al., 2017; Tudorascu et al., 2014; van Uden et al., 2015). Moreover, a recent study showed the association between microstructural integrity and depressive symptoms in a very large population (Shen et al., 2019).

Together, evidence for the associations between markers of cerebrovascular pathology or neurodegeneration and depression is accumulating but remains inconclusive. This might in part be due to differences across age. Evidence mostly comes from studies in elderly samples, but many of these brain changes might already start from middle age (Vinke et al., 2018). Additionally, evidence is based on mostly cross-sectional studies; however, longitudinal designs are needed to interpret temporal associations. Previous studies also focused mainly on a limited number of neuroimaging markers, comparing their effects on depression, therefore, remains difficult. In the current study, we assessed cross-sectional and longitudinal associations of multiple neuroimaging markers for vascular and neurodegenerative pathologies with depressive symptoms in middle-aged and elderly persons over time.

## Methods

### Study sample and design

This study was embedded in the Rotterdam Study, a prospective population-based cohort of middle-aged and elderly people, which started in Rotterdam, The Netherlands, in 1990 (Ikram et al., 2020). The overall aim of the study is to discover the causes and risk factors of age-related diseases. Between 2006 and 2012, 6087 participants were evaluated eligible for a magnetic resonance imaging (MRI) scan. Of those that agreed, 5051 had a gradable MRI scan. We further excluded 67 participants with a diagnosis of dementia and 41 participants with incomplete baseline data on depressive symptoms, leaving 4943 participants in the cross-sectional sample. Of those, follow-up data on depressive symptoms could be collected for 4103 participants. Depressive symptom assessments were conducted twice with a median follow-up time of 5.5 years (IQR = 0.9).

The Rotterdam Study has been approved by the Medical Ethics Committee of the Erasmus MC (registration number MEC 02.1015) and by the Dutch Ministry of Health, Welfare and Sport (Population Screening Act WBO, license number 1071272-159521-PG). The Rotterdam Study has been entered into the Netherlands National Trial Register (NTR; www.trialregister.nl) and into the WHO International Clinical Trials Registry Platform (ICTRP; www.who.int/ictrp/network/primary/en/) under shared catalog number NTR6831. All participants provided written informed consent to participate in the study and to have their information obtained from treating physicians.

### Measures

#### Neuroimaging markers

A brain MRI was performed with a single 1.5T MRI unit fitted with an eight-channel head coil (General Electric Healthcare, Milwaukee, USA, software version 11×). 3D T1-weighted, 3D T2-weighted gradient recalled echo, 2D proton-density weighted, 2D fluid-attenuated inversion recovery (FLAIR) images were included. For diffusion tensor imaging (DTI) measures; a single shot, diffusion-weighted spin echo-planar imaging sequence was used. Protocol details can be found elsewhere (Ikram et al., 2015).

Pre and post-processing steps have been explained elsewhere (Ikram et al., 2015). For brain tissue segmentation, the *k*-nearest-neighbor brain tissue method was used, with an extension for WMH classification (de Boer et al., 2009). Succinctly, image voxels were labeled as gray matter, white matter, WMH, cerebrospinal fluid (CSF) or background, and quantitative measures of volumes (in ml) were obtained. Total brain volume was represented as the sum of gray matter, normal-appearing white matter, and WMH. Intracranial volume (ICV) is the sum of total brain volume and CSF.

Lacunar infarcts were defined as focal lesions in size between 3 and 15 mm, showing the same signal intensity as CSF on T1-weighted image, T2-weighted image, and FLAIR sequence with a border of hyperintense signal (Hilal et al., 2019). Infarcts with the involvement of cortical gray matter were defined as cortical infarcts. Microbleeds were detected using three-dimensional T2-weighted gradient-recalled echo scans, they were determined as focal areas of very low signal intensity with the size of smaller than 10 mm (Vernooij et al., 2008). All lesions were graded by trained researchers who were blinded to clinical information.

In terms of DTI metrics, a standardized procedure was applied for data preprocessing, and estimation of FA and MD was performed in all voxels classified as normal-appearing white matter and averaged over these voxels to obtain mean values (Koppelmans et al., 2014). Lower levels of FA and higher levels of MD reflect reduced white matter microstructural integrity.

#### Depressive symptoms

To assess depressive symptoms, the validated Dutch version of the Center for Epidemiologic Studies Depression (CES-D) scale was used during the home interview (Beekman et al., 1997; Radloff, 1977). The CES-D is a self-report scale including 20 items with a total score range from 0 to 60. Higher scores reflect more depressive symptoms, with a total score of ≥16 indicating ‘clinically relevant depressive symptoms’. In terms of missing items in the scale, a weighted score was calculated if more than 75% of the items were completed. Otherwise, scores were set to missing.

#### Covariates

Age, sex, education, smoking status, alcohol consumption, body mass index (BMI), hypertension, type 2 diabetes, hyperlipidemia, heart diseases were included as covariates. Education was grouped into four categories as primary, low, intermediate, and high; as stated in the International Standard Classification of Education. Smoking status (never, former, current) was assessed during the home interview. The amount of alcohol consumption was determined during the home interview, calculated as gram/day. Height and weight were measured without shoes and heavy clothing on calibrated scales at the research center; to calculate BMI, weight (kg) divided by the squared height (m). Blood pressure was measured at the right brachial artery in a sitting position. The mean of two consecutive measurements was used. Hypertension was defined as resting blood pressure exceeding 140/90 mmHg or the use of blood pressure-lowering medication. Blood samples were taken during the research center visit. Type 2 diabetes was defined as either a fasting serum glucose concentration of ≥7.0 mmol/l or a non-fasting serum glucose concentration of ≥11.1 mmol/l or the use of glucose-lowering medications. The total cholesterol level was determined with an automated procedure. Hyperlipidemia was defined as the serum cholesterol concentration of ≥5.5 mmol/l or the use of lipid reducing medications. Heart diseases were defined as having a history of myocardial infarction or heart failure or atrial fibrillation or cardiac intervention (angioplasty, coronary artery bypass grafting, and other coronary revascularization procedures) based on medical records and information collected during the home interview. Anxiety disorders were determined with the Munich Version of Composite International Diagnostic Interview (M-CIDI) (Wittchen, Lachner, Wunderlich, & Pfister, 1998) and categorized as having at least one kind of anxiety disorder (yes/no).

#### Statistical analysis

Missing values for the covariates were less than 1.3% and were accounted for using multiple imputations (*n* = 15 imputed datasets), and we present the pooled results. We calculated standardized *z*-scores for the continuous variables indicating total brain volume, gray matter volume, white matter volume, WMH, FA, and MD. Cortical infarcts, lacunar infarcts, and microbleeds were used dichotomously (absence/presence). Depressive symptoms were analyzed as a continuous score. WMH and CES-D scores were log-transformed because of the skewed distribution.

Cross-sectional associations between neuroimaging markers and depressive symptoms at baseline were assessed with linear regression analyses. Results were analyzed in two models, model 1 adjusted for age and sex, and model 2 additionally adjusted for education, smoking status, alcohol consumption, BMI, hypertension, diabetes, hyperlipidemia, heart diseases. Analyses that included total brain volume, gray matter volume, white matter volume, WMH, FA, and MD were additionally adjusted for intracranial volume in both models to account for a potential influence of ICV. Separate models were conducted for each neuroimaging marker to prevent collinearity.

Linear mixed models were conducted including baseline neuroimaging markers and two repeated assessments of depressive symptoms to assess longitudinal associations. Adjustments for covariates were done per models 1 and 2, additionally including follow-up time and the interaction term between neuroimaging markers and follow-up time which describes the association between a neuroimaging marker and changes over time in depressive symptoms. Time between two examinations of depressive symptoms was used as follow-up time. Similarly, individual analyses were performed for each neuroimaging marker. A random intercept was included in all linear mixed models; time was included as a fixed effect.

As exploratory analyses aiming to examine any differences in associations across age groups, all analyses were repeated stratified for age groups. To do so the study sample was divided into three age groups, based on tertiles (i.e. aged ≤60, aged >60 and ≤70, and aged >70).

To prevent any effect of comorbid anxiety disorder, all analyses were repeated excluding participants who had any anxiety disorder. Additionally, analyses were repeated excluding those who had a cortical infarct, since cortical infarcts may have an effect on the calculation of brain tissue segmentation. Lastly, we analyzed the associations with dichotomized depressive symptoms using the cut-off score of 16 to assess the associations with the presence of clinically relevant depressive symptoms. We used logistic regression for cross-sectional analyses and generalized linear mixed models for the longitudinal associations. Since nine different neuroimaging markers were analyzed, we used false discovery rate correction and presented both unadjusted and adjusted *p* values (Glickman, Rao, & Schultz, 2014). All analyses were performed using R version 3.4.1. R packages mice, stats, lme4, and GLMMadaptive were used during analyses.

## Results

Characteristics of the study population are presented in Table 1. The mean age at baseline was 64.6 years (sd = 11.1) and 2755 participants (55.7%) were women. The median baseline depressive symptom score was 3 (IQR = 0–6), 9.2% of the population reported a score above the threshold indicating clinically relevant symptoms. The median follow-up duration between depression assessments was 5.5 years (IQR = 0.9). At the follow-up assessment, the median depressive symptoms score was 3 (IQR = 0–6).

**Table 1.** Descriptive characteristics of the study population (*N* = 4943)

	Mean ± SD
	N (%)
	Median [IQR]
Age, years	64.57 ± 11.12
Women	2755 (55.7)
Education	
Primary	438 (8.8)
Low	1864 (37.7)
Intermediate	1488 (30.1)
High	1112 (22.4)
Follow-up time, years	5.54 [4.70–5.57]
Total brain volume, ml	936.22 ± 100.98
Gray matter volume, ml	529.18 ± 54.68
White matter volume, ml	407.04 ± 60.41
White matter hyperintensities volume, ml	3.13 [0.59–5.67]
Cortical infarcts	179 (3.6)
Lacunar infarcts	390 (7.8)
Microbleeds	965 (19.5)
Fractional anisotropy^a^	0.33 ± 0.01
Mean diffusivity^a^, 10^−3^ mm^2^/s	0.74 ± 0.03
Baseline depressive symptom score	3 [0–6]
Baseline clinically significant depressive symptoms	458 (9.2)
Follow-up depressive symptom score^b^	3 [0–6]
Follow-up clinically significant depressive symptoms^b^	368 (7.4)
Smoking status	
Never smoked	1551 (31.3)
Former smoker	2392 (48.3)
Current smoker	1000 (20.2)
Alcohol consumption, g/day	6.42 [2.41–10.43]
BMI, kg/m^2^	27.42 ± 4.15
Hypertension	3192 (64.5)
Diabetes	650 (13.1)
Hyperlipidemia	3437 (69.5)
Heart diseases	539 (10.9)
Anxiety disorders	285 (5.7)

SD, standard deviation; IQR, interquartile range; BMI, body mass index.

a 4853 participants have data for DTI measures.

b 840 participants did not contribute data at the follow-up depression assessment.

The cross-sectional analyses showed that smaller total brain volume (*β* = −0.107, 95% CI −0.192 to −0.022), larger WMH volume (*β* = 0.047, 95% CI 0.010–0.084), presence of cortical infarcts (*β* = 0.194, 95% CI 0.047–0.341), and higher MD levels (*β* = 0.060, 95% CI 0.022–0.098) were associated with more depressive symptom scores in fully adjusted models (Table 2) after multiple testing correction. After stratification for age, the associations of larger WMH volume, presence of cortical infarcts and microbleeds, and higher MD levels with depressive symptoms were observed in persons aged above 70 years, and effect sizes were typically more pronounced in this older age group (online Supplementary Table S1). When those with an anxiety disorder were excluded (remaining sample size *n* = 4471), results were similar (data not shown). Equally, results for brain volumes, WMH volume, and DTI measures remained similar when participants with cortical infarcts were excluded (*n* = 4764, online Supplementary Table S2). Analyses with the dichotomized score indicating the presence of clinically relevant depressive symptoms only showed a statistically significant association between higher MD levels and clinically relevant depressive symptoms (OR 1.29, 95% CI 1.13–1.48, online Supplementary Table S3).

**Table 2.** Cross-sectional associations between neuroimaging markers and baseline depressive symptoms

	Depressive symptoms					
	Model 1			Model 2		
	*β* (95% CI)	*p* value	Adjusted *p* value*	*β* (95% CI)	*p* value	Adjusted *p* value*
Total brain volume	−0.142 (−0.226 to −0.058)	**<0.001**	**0.003**	−0.107 (−0.192 to −0.022)	**0.01**	**0.02**
Gray matter volume	−0.033 (−0.084 to 0.018)	0.20	0.22	−0.023 (−0.074 to 0.028)	0.37	0.42
White matter volume	−0.051 (−0.099 to −0.003)	**0.03**	**0.04**	−0.038 (−0.086 to 0.009)	0.11	0.17
WMH volume	0.061 (0.025 to 0.097)	**<0.001**	**0.003**	0.047 (0.010 to 0.084)	**0.01**	**0.02**
Cortical infarcts	0.231 (0.084 to 0.378)	**0.002**	**0.004**	0.194 (0.047 to 0.341)	**0.009**	**0.02**
Lacunar infarcts	0.123 (0.019 to 0.226)	**0.02**	**0.04**	0.096 (−0.007 to 0.200)	0.06	0.11
Microbleeds	0.044 (−0.027 to 0.116)	0.22	0.22	0.027 (−0.045 to 0.098)	0.46	0.46
Fractional anisotropy	−0.028 (−0.057 to 0.001)	0.06	0.08	−0.020 (−0.049 to 0.010)	0.19	0.24
Mean diffusivity	0.065 (0.027 to 0.103)	**<0.001**	**0.003**	0.060 (0.022 to 0.098)	**0.001**	**0.009**

CI, confidence interval; WMH, white matter hyperintensities.

Model 1 was adjusted for age and sex. Model 2 was adjusted for age, sex, education, smoking status, alcohol consumption, body mass index (BMI), hypertension, type 2 diabetes, hyperlipidemia, heart diseases. Intracranial volume was also included in both the two models for total brain volume, gray matter volume, white matter volume, WMH, fractional anisotropy, and mean diffusivity and *Z* scores were calculated for those variables. The *β* coefficient indicates the adjusted mean difference in CES-D score for every standard deviation change in each neuroimaging marker.

* False discovery rate adjusted *p* value.

Longitudinal analyses showed that smaller total brain volume (*β* = −0.091, 95% CI −0.167 to −0.015) and presence of cortical infarcts (*β* = 0.168, 95% CI 0.022–0.314) at baseline were associated with higher depressive symptom scores over time in multivariate adjusted models (Table 3). The interaction terms, which indicate the effect of the neuroimaging marker on changes over time in depressive symptoms, showed that with each standard deviation (SD) larger total brain volume, the yearly increase in depressive symptom score was reduced by 0.009 (95% CI −0.014 to −0.003, model 2). Additionally, with each SD higher white matter volume the yearly increase in depressive symptom score of 0.011 was diminished by 0.010 (95% CI −0.016 to −0.004, model 2) and every SD higher MD raised the yearly increase in depressive symptom score by 0.011 (95% CI 0.005–0.017, model 2). In exploratory analyses with stratification for age, larger WMH volume, presence of microbleeds, and higher MD levels were associated with more depressive symptoms over time in those aged above 70 years; effect sizes were more pronounced in the oldest group (online Supplementary Table S4). When those with an anxiety disorder were excluded (remaining sample size *n* = 4471), results were similar (data not shown). Equally, results for brain volumes, WMH volume, and DTI measures remained similar when participants with cortical infarcts were excluded (*n* = 4764, online Supplementary Table S5). Analyses with the dichotomized score indicating the presence of clinically relevant depressive symptoms did not suggest statistically significant association of any neuroimaging marker with clinically relevant depressive symptoms (online Supplementary Table S6).

**Table 3.** Longitudinal associations between neuroimaging markers and depressive symptoms

	Depressive symptoms					
	Model 1			Model 2		
	*β* (95% CI)	*p* value	Adjusted *p* value*	*β* (95% CI)	*p* value	Adjusted *p* value*
Total brain volume	−0.129 (−0.204 to −0.054)	**<0.001**	**0.003**	−0.091 (−0.167 to −0.015)	**0.01**	**0.02**
Time	0.010 (0.004 to 0.016)	**<0.001**	**0.003**	0.011 (0.005 to 0.016)	**<0.001**	**0.003**
Total brain volume × time	−0.009 (−0.015 to −0.003)	**0.002**	**0.004**	−0.009 (−0.014 to −0.003)	**0.002**	**0.005**
Gray matter volume	−0.010 (−0.057 to 0.036)	0.66	0.72	0.001 (−0.046 to 0.047)	0.98	0.98
Time	0.010 (0.004 to 0.015)	**<0.001**	**0.003**	0.010 (0.004 to 0.016)	**<0.001**	**0.003**
Gray matter volume × time	−0.005 (−0.011 to 0.001)	0.06	0.08	−0.005 (−0.011 to 0.001)	0.06	0.10
White matter volume	−0.040 (−0.084 to 0.003)	0.07	0.09	−0.027 (−0.071 to 0.016)	0.22	0.28
Time	0.010 (0.004 to 0.016)	**<0.001**	**0.003**	0.011 (0.005 to 0.016)	**<0.001**	**0.003**
White matter volume × time	−0.010 (−0.016 to −0.004)	**<0.001**	**0.003**	−0.010 (−0.016 to −0.004)	**<0.001**	**0.003**
WMH volume	0.038 (0.004 to 0.072)	**0.02**	**0.03**	0.022 (−0.012 to 0.056)	0.20	0.27
Time	0.010 (0.004 to 0.016)	**<0.001**	**0.003**	0.011 (0.005 to 0.017)	**<0.001**	**0.003**
WMH volume × time	0.006 (0.001 to 0.012)	**0.03**	0.05	0.006 (0.001 to 0.012)	**0.03**	0.05
Cortical infarcts	0.209 (0.063 to 0.356)	**0.005**	**0.01**	0.168 (0.022 to 0.314)	**0.02**	**0.03**
Time	0.009 (0.003 to 0.015)	**0.001**	**0.003**	0.010 (0.004 to 0.016)	**<0.001**	**0.003**
Cortical infarcts × time	−0.001 (−0.034 to 0.033)	0.96	0.96	−0.001 (−0.035 to 0.033)	0.96	0.98
Lacunar infarcts	0.095 (−0.007 to 0.198)	0.06	0.09	0.066 (−0.036 to 0.168)	0.20	0.27
Time	0.009 (0.003 to 0.015)	**0.002**	**0.004**	0.009 (0.004 to 0.015)	**0.001**	**0.003**
Lacunar infarcts × time	0.005 (−0.019 to 0.029)	0.67	0.72	0.005 (−0.018 to 0.029)	0.67	0.79
Microbleeds	0.022 (−0.049 to 0.092)	0.54	0.63	0.002 (−0.069 to 0.072)	0.95	0.98
Time	0.007 (0.001 to 0.013)	**0.02**	**0.03**	0.007 (0.001 to 0.014)	**0.01**	**0.02**
Microbleeds × time	0.013 (−0.001 to 0.029)	0.08	0.10	0.013 (−0.002 to 0.029)	0.08	0.12
Fractional anisotropy	−0.017 (−0.046 to 0.011)	0.23	0.28	−0.013 (−0.042 to 0.016)	0.38	0.47
Time	0.009 (0.003 to 0.015)	**0.001**	**0.003**	0.010 (0.004 to 0.016)	**<0.001**	**0.003**
Fractional anisotropy × time	−0.001 (−0.006 to 0.005)	0.88	0.91	−0.001 (−0.006 to 0.005)	0.85	0.96
Mean diffusivity	0.041 (0.006 to 0.077)	**0.02**	**0.03**	0.033 (−0.003 to 0.069)	0.06	0.10
Time	0.011 (0.005 to 0.017)	**<0.001**	**0.003**	0.012 (0.006 to 0.018)	**<0.001**	**0.003**
Mean diffusivity × time	0.010 (0.005 to 0.017)	**<0.001**	**0.003**	0.011 (0.005 to 0.017)	**<0.001**	**0.003**

CI, confidence interval; WMH, white matter hyperintensities.

Model 1 was adjusted for age and sex. Model 2 was adjusted for age, sex, education, smoking status, alcohol consumption, body mass index (BMI), hypertension, type 2 diabetes, hyperlipidemia, heart diseases. Intracranial volume was also included in both the two models for total brain volume, gray matter volume, white matter volume, WMH, fractional anisotropy, and mean diffusivity and *Z* scores were calculated for those variables.

The *β* coefficient indicates the adjusted mean difference in CES-D score for every standard deviation change in each neuroimaging marker. The *β* for multiplicative interaction term indicates the yearly increase in depressive symptoms with each standard deviation change in the respective neuroimaging marker.

* False discovery rate adjusted *p* value, *p* values below 0.05 are shown in bold.

## Discussion

In this population-based study, smaller total brain volume, larger WMH volume, presence of cortical infarcts, and higher levels of MD, indicating reduced white matter microstructural integrity, were associated with more depressive symptoms cross-sectionally. Total brain volume and cortical infarcts at baseline were also longitudinally associated with depressive symptoms after full confounder adjustment. A large total brain volume and white matter volume did reduce the yearly increase of depressive symptoms, when taking time into account. Moreover, high MD levels were also shown to raise the yearly increase of depressive symptoms over time.

Our study suggests that higher MD levels are associated with depressive symptoms cross-sectionally and longitudinally in this population of middle-aged and elderly persons. Higher MD levels are also associated with clinically relevant depressive symptoms cross-sectionally, but not over time. This might be explained by a lack of statistical power but could also suggest the importance of neuroimaging markers on subclinical symptoms in this population-based sample of middle-aged and elderly persons. The lack of associations for FA in this study could be explained by the idea that MD is more reproducible than FA across the entire brain (Luque Laguna et al., 2020). Still, our findings are in line with previous studies suggesting white matter structures play an important role in depression (Brookes et al., 2014; Charlton et al., 2014; Qiu et al., 2017; Reppermund et al., 2014; Shen et al., 2017, 2019; van Uden et al., 2015; Xiao, He, Mcwhinnie, & Yao, 2015). Different neuronal circuits are involved in mood regulation and cognitive processes (Kanske, Heissler, Schonfelder, & Wessa, 2012; Snyder, 2013) and the role of these circuits on depression has been discussed for a long time (Alexopoulos, 2002; Taylor et al., 2003). It is widely accepted that disintegration of frontal, frontostriatal, and fronto-limbic networks are in part responsible for the occurrence of late-life depression (Alexopoulos, 2019; Wen, Steffens, Chen, & Zainal, 2014; Zhang, Peng, Sweeney, Jia, & Gong, 2018), thus damages of white matter structures might be crucial in the pathogenesis of late-life depression. These pathological transformations have also been defined together as the ‘disconnection syndrome’ (Liao et al., 2013). Results from the current study seem to support this hypothesis positing the importance of microstructural architecture in the etiopathogenesis of late-life depression. Notably, exploratory analyses showed effect sizes most pronounced at old age, which could be due to the more common occurrence of health conditions, including cardiovascular disease, that affect white matter integrity at old age. Nevertheless, the results on different age groups should be assessed carefully since stratified analyses could result in reduced statistical power.

Our longitudinal results indicate that smaller brain volume is associated with depressive symptoms, and not only smaller brain volume but also smaller white matter volume were related to the rate of increase of depressive symptoms over time in this population of middle-aged and elderly persons. This clearly reflects the effects of brain volume and white matter volume on changes over time in depressive symptoms. Although these associations of brain volumes and depression were established by some studies before (Grool et al., 2012; Qiu et al., 2017; van Sloten et al., 2015), the exact biological mechanism responsible for the associations remains unknown. Cerebrovascular lesions are one of the important reasons for brain volume reductions, and these lesions that underlie the differences in brain volumes could be partly responsible for the change in depressive symptoms over time. Cerebrovascular lesions trigger biological processes including vascular changes, HPA axis dysregulation, inflammation, amyloid deposition, tissue repair dysregulations, disruption of allostasis, or genetic and epigenetic influences, and these alterations may predispose or induce depression (Alexopoulos, 2019), yet we did not find specific associations of focal vascular lesions with depressive symptoms in our study. Of note, white matter volume and MD were not related to higher depressive symptoms *per se*, they were found to be related to the increased rate of symptoms over time, which could suggest that the assessed pathologies are just one of many contributing factors within various overlapping neurobiological mechanisms underlying depression.

Cerebrovascular pathology has been the main focus of the literature on late-life depression and vascular depression, and its role has been indicated before (Aizenstein et al., 2016; van Agtmaal et al., 2017). On the other hand, lacunar infarcts and cerebral microbleeds were not found to be related to depression in a meta-analysis of longitudinal studies (Fang et al., 2020). In our study, we found longitudinal associations of cortical infarcts with depressive symptoms and larger WMH volume and the yearly increase of depressive symptoms before multiple testing correction. Again, we found no association between CSVD markers and clinically relevant depressive symptoms over time. Our results on cerebrovascular pathology and depressive symptoms could be explained in multiple ways. First, CSVD stems from many histopathological changes, and these changes are known to have perilesional and remote effects via neuronal fibers (Ter Telgte et al., 2018). Traditional CSVD imaging markers (WMH, lacunes, etc.) are focal but there is an increasing insight that there are more widespread changes in the brain resulting from CSVD, which are better captured by global markers such as white matter atrophy or DTI measures. Although there might not be any observable changes in these conventional MRI markers in our study, DTI metrics might still be affected (Maillard et al., 2014) as these have been suggested to reflect brain pathology more sensitively than the conventional focal markers of CSVD (Lawrence et al., 2013; Tuladhar et al., 2015). Our results suggest that this might also be true for late-life depressive symptoms in population-based samples. Second, the absence of an association of the traditional focal CSVD markers with depressive symptoms in our study might also be explained by including middle-aged persons in our study. The majority of the studies examining the relations of cerebrovascular pathology and depression were performed in elderly populations and brain alterations and depression are known to be increased by age (Mitchell & Subramaniam, 2005; Pedersen et al., 2014; Vinke et al., 2018). Recent findings from the Maastricht Study showed significant associations of WMHs and depressive symptoms in only older age groups (Geraets et al., 2021) and our exploratory age-stratified analyses posit the same direction, effect sizes were more pronounced in older ages. This may suggest that cerebrovascular lesions accumulate over time and cause depression when they reach a certain threshold, which is not commonly reached at middle-age, or that the pathology is not common enough at middle-age. Third, the current study focuses on depressive symptoms, independent of whether these can be classified as a depressive disorder. Subclinical depressive symptoms particularly burden older individuals which might not meet the criteria for clinical diagnosis but still suffer from everyday impact. Lastly, the heterogeneous nature of depression pathogenesis and limitations for the applications of the strict vascular depression criteria restrain the generalizability of the associations between CSVD and late-life depression (Steffens, 2019).

The present study has several potential limitations. First, we had neuroimaging data solely at baseline. Additionally, we only measured depressive symptoms at two time points and had no information on depressive symptoms in between these two time points, limiting our possibilities to take into account the fluctuating nature of depressive symptoms. Second, we did not diagnose depressive disorders with a clinical interview but instead used a validated questionnaire to assess depressive symptoms. This does however ensure that we are able to capture subclinical depressive symptoms, which are common in middle-aged and elderly persons. Third, we did not define vascular depression specifically, hence we can only extrapolate from our findings on late-life depression. Yet, the present study also has several strengths, we used a large sample size drawn from the general population including middle-aged and elderly persons, used a longitudinal design assessing repeated measures of depressive symptoms and assessed a multitude of brain imaging markers within one population.

## Conclusion

The current study supports the role of pathological brain alterations on the occurrence of late-life depression; in particular of smaller total brain volume, smaller white matter volume, and reduced integrity of white matter. White matter integrity appeared to be the most consistently related factor for having depressive symptoms in middle-aged and elderly individuals over time. Using repeated measures of neuroimaging markers and targeting graph measures of brain connectivity in the future will further the understanding of the temporal role of white matter microstructure in depression.

## Supplementary material

For supplementary material accompanying this paper visit https://doi.org/10.1017/S003329172200112X. click here to view supplementary material
