# Supplementary material for: Associations of neuroimaging markers with depressive symptoms over time in middle-aged and elderly persons
Source: Psychol Med. 2022 May 10;53(10):4355–63. doi: 10.1017/S003329172200112X (PMC10388307; doi:10.1017/S003329172200112X)
Supplement: Supplementary file 1 [file S003329172200112Xsup001.docx]

**Supplemental Tables**

Table S1: Cross-sectional associations between neuroimaging markers and baseline depressive symptoms in different age groups

|  | Depressive symptoms (Model 2) | | | | | | | | | |
| --- | --- | --- | --- | --- | --- | --- | --- | --- | --- | --- |
|  | Aged < 60 (n=2115) | |  | Aged between  60 – 70 (n=1072) | |  | | Aged > 70 (n=1756) | | |
|  | beta (95% CI) | p value | Adjusted  p value* | beta (95% CI) | p value | Adjusted  p value* | beta (95% CI) | | p value | Adjusted  p value* |
| Total brain volume | -0.023 (-0.159 ; 0.113) | 0.73 | 0.76 | -0.097 (-0.278 ; 0.085) | 0.29 | 0.56 | -0.145 (-0.295 ; 0.004) | | 0.05 | 0.09 |
| Gray matter volume | -0.053 (-0.134 ; 0.028) | 0.20 | 0.76 | 0.073 (-0.041 ; 0.187) | 0.21 | 0.56 | -0.042 (-0.125 ; 0.042) | | 0.32 | 0.36 |
| White matter volume | 0.033 (-0.047 ; 0.112) | 0.41 | 0.76 | -0.112 (-0.218 ; 0.006) | 0.03 | 0.27 | -0.035 (-0.114 ; 0.045) | | 0.39 | 0.39 |
| WMH volume | 0.037 (-0.027 ; 0.101) | 0.25 | 0.76 | 0.012 (-0.066 ; 0.090) | 0.76 | 0.83 | 0.075 (0.021 ; 0.128) | | **0.006** | **0.03** |
| Cortical infarcts | 0.045 (-0.250 ; 0.340) | 0.76 | 0.76 | 0.265 (-0.075 ; 0.605) | 0.12 | 0.54 | 0.230 (0.027 ; 0.433) | | **0.02** | **0.04** |
| Lacunar infarcts | 0.042 (-0.195 ; 0.280) | 0.72 | 0.76 | 0.117 (-0.122 ; 0.356) | 0.33 | 0.56 | 0.092 (-0.042 ; 0.227) | | 0.17 | 0.22 |
| Microbleeds | -0.031 (-0.169 ; 0.107) | 0.65 | 0.76 | -0.026 (-0.179 ; 0.126) | 0.73 | 0.83 | 0.123 (0.020 ; 0.226) | | **0.01** | **0.03** |
| Fractional anisotropy | -0.014 (-0.064 ; 0.036) | 0.57 | 0.76 | -0.007 (-0.072 ; 0.058) | 0.83 | 0.83 | -0.032 (-0.078 ; 0.014) | | 0.17 | 0.22 |
| Mean diffusivity | 0.013 (-0.056 ; 0.083) | 0.70 | 0.76 | 0.036 (-0.044 ; 0.116) | 0.37 | 0.56 | 0.074 (0.017 ; 0.132) | | **0.01** | **0.03** |
|  |  |  |  |  |  |  |  | |  |  |

*False discovery rate adjusted p values

OR: odds ratio, CI: confidence interval, WMH: white matter hyperintensities

Model 1 was adjusted for age and sex. Model 2 adjusted for age, sex, education, smoking status, alcohol consumption, body mass index (BMI), hypertension,

type 2 diabetes, hyperlipidemia, heart diseases. Intracranial volume was also included in both two models for total brain volume, gray matter volume, white matter volume,

WMH, fractional anisotropy and mean diffusivity and Z scores were calculated for those variables. The beta coefficient indicates the adjusted mean difference in CES-D

score for every standard deviation change in each neuroimaging marker.

| Table S2: Cross-sectional associations between neuroimaging markers and baseline depressive symptoms (participants without cortical infarcts) | | | | | |  |
| --- | --- | --- | --- | --- | --- | --- |
|  |  | Depressive symptoms | | | |  |
|  |  |  |  |  |  |  |
|  | Model 1 |  |  | Model 2 |  |  |
|  | beta (95% CI) | p value | Adjusted  p value* | beta (95% CI) | p value | Adjusted  p value* |
| Total brain volume | -0.122 (-0.210 ; -0.034) | **0.006** | **0.02** | -0.081 (-0.171 ; 0.009) | 0.07 | 0.19 |
| Gray matter volume | -0.030 (-0.082 ; 0.022) | 0.26 | 0.26 | -0.021 (-0.074 ; 0.032) | 0.43 | 0.43 |
| White matter volume | -0.040 (-0.089 ; 0.009) | 0.11 | 0.15 | -0.025 (-0.074 ; 0.025) | 0.33 | 0.38 |
| WMH volume | 0.060 (0.023 ; 0.098) | **0.001** | **0.008** | 0.048 (0.009 ; 0.086) | **0.01** | **0.04** |
| Lacunar infarcts | 0.101 (-0.010 ; 0.212) | 0.07 | 0.14 | 0.079 (-0.033 ; 0.192) | 0.17 | 0.34 |
| Microbleeds | 0.048 (-0.025 ; 0.121) | 0.20 | 0.23 | 0.045 (-0.027 ; 0.118) | 0.22 | 0.35 |
| Fractional anisotropy | -0.026 (-0.056 ; 0.004) | 0.09 | 0.14 | -0.017 (-0.048 ; 0.014) | 0.27 | 0.36 |
| Mean diffusivity | 0.061 (0.022 ; 0.100) | **0.002** | **0.008** | 0.055 (0.016 ; 0.095) | **0.006** | **0.04** |
|  |  |  |  |  |  |  |

* False discovery rate adjusted p values

CI: confidence interval, WMH: white matter hyperintensities

Model 1 was adjusted for age and sex. Model 2 adjusted for age, sex, education, smoking status, alcohol consumption,

body mass index (BMI), hypertension, type 2 diabetes, hyperlipidemia, heart diseases. Intracranial volume was also included in both

two models for total brain volume, gray matter volume, white matter volume, WMH, fractional anisotropy and mean diffusivity and

Z scores were calculated for those variables. The beta coefficient indicates the adjusted mean difference in CES-D score for every

standard deviation change in each neuroimaging marker.

| Table S3: Cross-sectional associations between neuroimaging markers and baseline clinically significant depressive symptoms | | | | | |  |
| --- | --- | --- | --- | --- | --- | --- |
|  |  | Clinically significant depressive symptoms | | | |  |
|  |  |  |  |  |  |  |
|  | Model 1 |  |  | Model 2 |  |  |
|  | OR (95% CI) | p value | Adjusted  p value* | OR (95% CI) | p value | Adjusted  p value* |
| Total brain volume | 0.71 (0.53 ; 0.95) | **0.02** | **0.04** | 0.76 (0.55 ; 1.03) | 0.08 | 0.18 |
| Gray matter volume | 0.89 (0.74 ; 1.07) | 0.23 | 0.32 | 0.91 (0.75 ; 1.11) | 0.38 | 0.49 |
| White matter volume | 0.90 (0.76 ; 1.07) | 0.25 | 0.32 | 0.92 (0.76 ; 1.10) | 0.36 | 0.49 |
| WMH volume | 1.20 (1.06 ; 1.36) | **0.003** | **0.009** | 1.14 (1.01 ; 1.30) | **0.03** | 0.09 |
| Cortical infarcts | 1.47 (0.92 ; 2.35) | 0.09 | 0.16 | 1.36 (0.84 ; 2.20) | 0.20 | 0.36 |
| Lacunar infarcts | 1.16 (0.82 ; 1.66) | 0.38 | 0.43 | 1.06 (0.74 ; 1.53) | 0.72 | 0.72 |
| Microbleeds | 1.04 (0.81 ; 1.33) | 0.74 | 0.74 | 1.05 (0.81 ; 1.37) | 0.67 | 0.72 |
| Fractional anisotropy | 0.84 (0.76 ; 0.93) | **0.001** | **0.004** | 0.87 (0.78 ; 0.97) | **0.01** | 0.05 |
| Mean diffusivity | 1.30 (1.15 ; 1.48) | **<0.001** | **0.004** | 1.29 (1.13 ; 1.48) | **<0.001** | **0.009** |
|  |  |  |  |  |  |  |

* False discovery rate adjusted p values

OR: odds ratio, CI: confidence interval, WMH: white matter hyperintensities

Model 1 was adjusted for age and sex. Model 2 adjusted for age, sex, education, smoking status, alcohol consumption,

body mass index (BMI), hypertension, type 2 diabetes, hyperlipidemia, heart diseases. Intracranial volume was also included in both

two models for total brain volume, gray matter volume, white matter volume, WMH, fractional anisotropy and mean diffusivity and

Z scores were calculated for those variables.

| Table S4: Longitudinal associations between neuroimaging markers and depressive symptoms in different age groups | | | | | | | | | | | | | | | | | |
| --- | --- | --- | --- | --- | --- | --- | --- | --- | --- | --- | --- | --- | --- | --- | --- | --- | --- |
|  |  | Depressive symptoms (Model 2) | | | | | | | |  |  | | |  |  | | |
|  | Aged < 60  (n=2,115) |  | |  | | Aged between  60 – 70 (n=1,072) |  | | |  | Aged > 70  (n=1,756) | | |  |  | | |
|  | beta (95% CI) | p value | Adjusted  p value* | | beta (95% CI) | | p value | | Adjusted  p value* |  | beta (95% CI) | | | p value | Adjusted  p value* | | |
| Total brain volume | -0.004 (-0.123 ; 0.115) | 0.94 | 0.96 | | -0.091 (-0.250 ; 0.069) | | | 0.26 | 0.81 | | | -0.137 (-0.273 ; -0.001) | | **0.04** | 0.08 | | |
| Time | -0.001 (-0.009 ; 0.008) | 0.96 | 0.96 | | -0.004 (-0.016 ; 0.009) | | | 0.56 | 0.81 | | | 0.040 (0.028 ; 0.052) | | **<0.001** | **0.003** | | |
| Total brain volume*Time | -0.005 (-0.013 ; 0.004) | 0.27 | 0.96 | | -0.004 (-0.018 ; 0.009) | | | 0.53 | 0.81 | | | -0.001 (-0.013 ; 0.010) | | 0.80 | 0.83 | | |
|  |  |  |  | |  | | |  |  | | |  | |  |  | | |
| Gray matter volume | -0.023 (-0.096 ; 0.049) | 0.52 | 0.96 | | 0.080 (-0.023 ; 0.182) | | | 0.12 | 0.81 | | | -0.047 (-0.125 ; 0.031) | | 0.23 | 0.37 | | |
| Time | -0.001 (-0.010 ; 0.007) | 0.76 | 0.96 | | -0.004 (-0.017 ; 0.008) | | | 0.51 | 0.81 | | | 0.040 (0.029 ; 0.051) | | **<0.001** | **0.003** | | |
| Gray matter volume*Time | -0.002 (-0.010 ; 0.006) | 0.65 | 0.96 | | -0.002 (-0.016 ; 0.011) | | | 0.74 | 0.87 | | | -0.002 (-0.013 ; 0.009) | | 0.68 | 0.77 | | |
|  |  |  |  | |  | | |  |  | | |  | |  |  | | |
| White matter volume | 0.032 (-0.039 ; 0.104) | 0.37 | 0.96 | | -0.103 (-0.199 ; -0.008) | | | **0.03** | 0.81 | | | -0.023 (-0.098 ; 0.050) | | 0.53 | 0.68 | | |
| Time | 0.001 (-0.008 ; 0.009) | 0.96 | 0.96 | | -0.003 (-0.016 ; 0.009) | | | 0.60 | 0.81 | | | 0.041 (0.029 ; 0.053) | | **<0.001** | **0.003** | | |
| White matter volume*Time | -0.006 (-0.015; 0.002) | 0.15 | 0.96 | | -0.005 (-0.019 ; 0.008) | | | 0.45 | 0.81 | | | -0.001 (-0.012 ; 0.012) | | 0.99 | 0.99 | | |
|  |  |  |  | |  | | |  |  | | |  | |  |  | | |
| WMH volume | 0.031 ( -0.031 ; 0.093) | 0.33 | 0.96 | | 0.016 (-0.060 ; 0.091) | | | 0.68 | 0.83 | | | 0.071 (0.019 ; 0.124) | | **0.008** | **0.02** | | |
| Time | -0.007 (-0.017 ; 0.004) | 0.22 | 0.96 | | -0.004 (-0.017 ; 0.009) | | | 0.54 | 0.81 | | | 0.043 (0.031 ; 0.057) | | **<0.001** | **0.003** | | |
| WMH volume*Time | -0.008 (-0.020 ; 0.004) | 0.17 | 0.96 | | 0.001 (-0.016 ; 0.016) | | | 0.97 | 0.97 | | | -0.004 (-0.016 ; 0.007) | | 0.45 | 0.64 | | |
|  |  |  |  | |  | | |  |  | | |  | |  |  | | |
| Cortical infarcts | 0.043 (-0.248 ; 0.335) | 0.76 | 0.96 | | 0.237 (-0.099 ; 0.574) | | | 0.16 | 0.81 | | | 0.220 (0.019 ; 0.422) | | **0.03** | | 0.06 |  |
| Time | -0.002 (-0.010 ; 0.006) | 0.66 | 0.96 | | -0.004 (-0.017 ; 0.008) | | | 0.50 | 0.81 | | | 0.042 (0.031 ; 0.053) | | **<0.001** | | **0.003** |  |
| Cortical infarcts*Time | -0.004 (-0.065 ; 0.056) | 0.88 | 0.96 | | 0.008 (-0.069 ; 0.085) | | | 0.84 | 0.87 | | | -0.028 (-0.077 ; 0.022) | | 0.27 | | 0.40 |  |
|  |  |  |  | |  | | |  |  | | |  | |  | |  |  |
| Lacunar infarcts | 0.048 (-0.187 ; 0.283) | 0.68 | 0.96 | | 0.094 (-0.143 ; 0.330) | | | 0.43 | 0.81 | | | 0.082 (-0.051 ; 0.216) | | 0.22 | | 0.36 |  |
| Time | -0.002 (-0.010 ; 0.006) | 0.68 | 0.96 | | -0.004 (-0.017 ; 0.009) | | | 0.57 | 0.81 | | | 0.041 (0.030 ; 0.053) | | **<0.001** | | **0.003** |  |
| Lacunar infarcts*Time | -0.006 (-0.054 ; 0.042) | 0.81 | 0.96 | | -0.006 (-0.059 ; 0.047) | | | 0.82 | 0.87 | | | -0.004 (-0.036 ; 0.028) | | 0.80 | | 0.83 |  |
|  |  |  |  | |  | | |  |  | | | |  |  | |  |  |
| Microbleeds | -0.035 (-0.171 ; 0.101) | 0.61 | 0.96 | | -0.063 (-0.215 ; 0.088) | | | 0.41 | 0.81 | | | | 0.114 (0.012 ; 0.215) | **0.02** | | **0.04** |  |
| Time | -0.003 (-0.012 ; 0.005) | 0.44 | 0.96 | | -0.006 (-0.020 ; 0.008) | | | 0.40 | 0.81 | | | | 0.043 (0.031 ; 0.056) | **<0.001** | | **0.003** |  |
| Microbleeds*Time | 0.013 (-0.013 ; 0.039) | 0.32 | 0.96 | | 0.010 (-0.024 ; 0.044) | | | 0.55 | 0.81 | | | | -0.008 (-0.031 ; 0.015) | 0.51 | | 0.67 |  |
|  |  |  |  | |  | | |  |  | | | |  |  | |  |  |
| Fractional anisotropy | -0.016 (-0.064 ; 0.033) | 0.52 | 0.96 | | -0.006 (-0.070 ; 0.058) | | | 0.84 | 0.87 | | | | -0.033 (-0.079 ; 0.013) | 0.16 | | 0.29 |  |
| Time | -0.003 (-0.011 ; 0.005) | 0.47 | 0.96 | | -0.005 (-0.018 ; 0.007) | | | 0.40 | 0.81 | | | | 0.042 (0.031 ; 0.053) | **<0.001** | | **0.003** |  |
| Fractional anisotropy*Time | 0.004 (-0.004 ; 0.014) | 0.32 | 0.96 | | 0.003 (-0.011 ; 0.017) | | | 0.64 | 0.82 | | | | 0.002 (-0.008 ; 0.013) | 0.65 | | 0.76 |  |
|  |  |  |  | |  | | |  |  | | | |  |  | |  |  |
| Mean diffusivity | 0.007 (-0.061 ; 0.076) | 0.83 | 0.96 | | 0.033 (-0.044 ; 0.111) | | | 0.39 | 0.81 | | | | 0.069 (0.012 ; 0.125) | **0.01** | | **0.02** |  |
| Time | -0.002 (-0.014 ; 0.009) | 0.70 | 0.96 | | -0.004 (-0.017 ; 0.009) | | | 0.54 | 0.81 | | | | 0.044 (0.031 ; 0.058) | **<0.001** | | **0.003** |  |
| Mean diffusivity*Time | -0.001 (-0.014 ; 0.013) | 0.93 | 0.96 | | 0.007 (-0.009 ; 0.023) | | | 0.40 | 0.81 | | | | -0.004 (-0.016 ; 0.008) | 0.55 | | 0.67 |  |
|  |  |  |  | | |  |  | | |  |  | | |  |  | | |

*False discovery rate adjusted p values

OR: odds ratio, CI: confidence interval, WMH: white matter hyperintensities

Model 1 was adjusted for age and sex. Model 2 adjusted for age, sex, education, smoking status, alcohol consumption, body mass index (BMI), hypertension, type 2 diabetes, hyperlipidemia, heart diseases. Intracranial volume was also included in both two models for total brain volume, gray matter volume, white matter volume, WMH, fractional anisotropy and mean diffusivity and Z scores were calculated for those variables.

The beta coefficient indicates the adjusted mean difference in CES-D score for every standard deviation change in each neuroimaging marker. Betas for multiplicative interaction terms indicate the yearly increase in depressive symptoms with each standard deviation change in the respective neuroimaging marker..

| Table S5: Longitudinal associations between neuroimaging markers and depressive symptom scores (participants without cortical infarcts) | | | | | | |  |
| --- | --- | --- | --- | --- | --- | --- | --- |
|  |  |  | | | Depressive symptoms |  |  |
|  | Model 1 |  |  | Model 2 | |  |  |
|  | beta (95% CI) | p value | Adjusted  p value* | beta (95% CI) | | p value | Adjusted  p value* |
| Total brain volume | -0.111 (-0.190 ; -0.033) | **0.005** | **0.01** | -0.072 (-0.152 ; 0.008) | | 0.07 | 0.12 |
| Time | 0.010 (0.004 ; 0.016) | **<0.001** | **0.003** | 0.009 (0.003 ; 0.015) | | **0.001** | **0.004** |
| Total brain volume*Time | -0.010 (-0.015 ; -0.004) | **0.001** | **0.003** | -0.009 (-0.015 ; -0.003) | | **0.002** | **0.007** |
|  |  |  |  |  | |  |  |
| Gray matter volume | -0.006 (-0.053 ; 0.042) | 0.81 | 0.81 | 0.002 (-0.045 ; 0.050) | | 0.92 | 0.92 |
| Time | 0.010 (0.004 ; 0.015) | **0.001** | **0.003** | 0.009 (0.003 ; 0.015) | | **0.003** | **0.009** |
| Gray matter volume*Time | -0.006 (-0.012 ; -0.001) | **0.04** | 0.06 | -0.005 (-0.011 ; 0.001) | | 0.06 | 0.11 |
|  |  |  |  |  | |  |  |
| White matter volume | -0.030 (-0.075 ; 0.015) | 0.19 | 0.19 | -0.016 (-0.061 ; 0.030) | | 0.49 | 0.57 |
| Time | 0.010 (0.004 ; 0.016) | **<0.001** | **<0.001** | 0.009 (0.003 ; 0.015) | | **0.001** | **0.004** |
| White matter volume*Time | -0.011 (-0.017 ; -0.005) | **<0.001** | **<0.001** | -0.010 (-0.016 ; -0.004) | | **<0.001** | **0.004** |
|  |  |  |  |  | |  |  |
| WMH volume | 0.035 (0.001 ; 0.071) | **0.04** | **0.06** | 0.023 (-0.013 ; 0.059) | | 0.21 | 0.30 |
| Time | 0.011 (0.005 ; 0.017) | **<0.001** | **0.003** | 0.010 (0.004 ; 0.016) | | **0.001** | **0.004** |
| WMH volume*Time | 0.007 (0.001 ; 0.013) | **0.03** | 0.06 | 0.006 (-0.001 ; 0.012) | | 0.06 | 0.11 |
|  |  |  |  |  | |  |  |
| Lacunar infarcts | 0.072 (-0.038 ; 0.182) | 0.20 | 0.25 | 0.050 (-0.062 ; 0.161) | | 0.38 | 0.48 |
| Time | 0.009 (0.003 ; 0.015) | **0.004** | **0.01** | 0.008 (0.002 ; 0.014) | | **0.01** | **0.02** |
| Lacunar infarcts*Time | 0.013 (-0.013 ; 0.038) | 0.33 | 0.39 | 0.012 (-0.013 ; 0.038) | | 0.34 | 0.45 |
|  |  |  |  |  | |  |  |
| Microbleeds | 0.025 (-0.047 ; 0.097) | 0.50 | 0.54 | 0.010 (-0.063 ; 0.083) | | 0.79 | 0.82 |
| Time | 0.007 (0.001 ; 0.013) | **0.03** | 0.06 | 0.006 (-0.001 ; 0.013) | | 0.06 | 0.11 |
| Microbleeds*Time | 0.014 (-0.001 ; 0.030) | 0.07 | 0.10 | 0.014 (-0.002 ; 0.029) | | 0.08 | 0.13 |
|  |  |  |  |  | |  |  |
| Fractional anisotropy | -0.014 (-0.044 ; 0.015) | 0.34 | 0.39 | -0.010 (-0.041 ; 0.020) | | 0.50 | 0.57 |
| Time | 0.009 (0.003 ; 0.015) | **0.002** | **0.005** | 0.008 (0.002 ; 0.014) | | **0.005** | **0.01** |
| Fractional anisotropy*Time | -0.001 (-0.006 ; 0.005) | 0.80 | 0.81 | -0.001 (-0.007 ; 0.005) | | 0.78 | 0.82 |
|  |  |  |  |  | |  |  |
| Mean diffusivity | 0.036 (-0.001 ; 0.072) | 0.05 | 0.07 | 0.030 (-0.007 ; 0.067) | | 0.11 | 0.17 |
| Time | 0.012 (0.006 ; 0.018) | **<0.001** | **0.003** | 0.011 (0.004 ; 0.017) | | **<0.001** | **0.004** |
| Mean diffusivity*Time | 0.012 (0.00 ; 0.018) | **<0.001** | **0.003** | 0.011 (0.004 ; 0.018) | | **<0.001** | **0.004** |
|  |  |  |  |  | |  |  |

*False discovery rate adjusted p values

CI: confidence interval, WMH: white matter hyperintensities

Model 1 was adjusted for age and sex. Model 2 adjusted for age, sex, education, smoking status, alcohol consumption, body mass index (BMI),

hypertension, type 2 diabetes, hyperlipidemia, heart diseases. Intracranial volume was also included in both two models for total brain volume,

gray matter volume, white matter volume, WMH, fractional anisotropy and mean diffusivity and Z scores were calculated for those variables.

The beta coefficient indicates the adjusted mean difference in CES-D score for every standard deviation change in each neuroimaging marker.

Betas for multiplicative interaction terms indicate the yearly increase in depressive symptoms with each standard deviation change in the respective

neuroimaging marker.

| Table S6 : Longitudinal associations between neuroimaging markers and clinically significant depressive symptoms | | | | | | |  |
| --- | --- | --- | --- | --- | --- | --- | --- |
|  |  | Clinically significant depressive symptoms | | | | |  |
|  | Model 1 | |  |  | Model 2 |  |  |
|  | OR (95% CI) | | p value | Adjusted  p value* | OR (95% CI) | p value | Adjusted  p value* |
| Total brain volume | 0.97 (0.57 ; 1.65) | | 0.93 | 0.99 | 0.84 (0.54 ; 1.31) | 0.46 | 0.78 |
| Time | 1.11 (0.95 ; 1.30) | | 0.16 | 0.46 | 1.11 (0.94 ; 1.32) | 0.20 | 0.66 |
| Total brain volume*Time | 0.97 (0.82 ; 1.14) | | 0.75 | 0.88 | 0.94 (0.80 ; 1.11) | 0.53 | 0.80 |
|  |  | |  |  |  |  |  |
| Gray matter volume | 0.93 (0.66 ; 1.30) | | 0.69 | 0.88 | 0.97 (0.71 ; 1.33) | 0.87 | 0.95 |
| Time | 1.11 (0.95 ; 1.30) | | 0.17 | 0.46 | 1.12 (0.94 ; 1.32) | 0.18 | 0.66 |
| Gray matter volume*Time | 1.04 (0.88 ; 1.24) | | 0.60 | 0.81 | 1.01 (0.85 ; 1.18) | 0.93 | 0.95 |
|  |  | |  |  |  |  |  |
| White matter volume | 1.04 (0.77 ; 1.42) | | 0.75 | 0.88 | 0.90 (0.66 ; 1.21) | 0.49 | 0.78 |
| Time | 1.11 (0.95 ; 1.30) | | 0.15 | 0.46 | 1.11 (0.94 ; 1.32) | 0.19 | 0.66 |
| White matter volume*Time | 0.92 (0.78 ; 1.08) | | 0.30 | 0.49 | 0.90 (0.77 ; 1.07) | 0.25 | 0.66 |
|  |  | |  |  |  |  |  |
| WMs volume | 0.89 (0.71 ; 1.11) | | 0.30 | 0.49 | 0.94 (0.73 ; 1.20) | 0.64 | 0.91 |
| Time | 1.10 (0.94 ; 1.29) | | 0.20 | 0.47 | 1.10 (0.91 ; 1.32) | 0.32 | 0.66 |
| WMH volume*Time | 0.91 (0.78 ; 1.06) | | 0.25 | 0.49 | 0.90 (0.75 ;1.08) | 0.30 | 0.66 |
|  |  | |  |  |  |  |  |
| Cortical infarcts | 1.70 (0.60 ; 4.84) | | 0.31 | 0.49 | 1.34 (0.67 ; 2.70) | 0.40 | 0.73 |
| Time | 1.11 (0.96 ; 1.30) | | 0.14 | 0.46 | 1.12 (0.94 ; 1.32) | 0.18 | 0.66 |
| Cortical infarcts*Time | 0.99 (0.39 ; 2.50) | | 0.99 | 0.99 | 0.98 ( 0.51 ; 1.86) | 0.95 | 0.95 |
|  |  | |  |  |  |  |  |
| Lacunar infarcts | 0.67 (0.33 ; 1.38) | | 0.28 | 0.49 | 0.96 (0.56 ; 1.67) | 0.91 | 0.95 |
| Time | 1.11 (0.95 ; 1.30) | | 0.15 | 0.46 | 1.12 (0.94 ; 1.33) | 0.17 | 0.66 |
| Lacunar infarcts*Time | 0.98 (0.53 ; 1.81) | | 0.95 | 0.99 | 0.90 (0.53 ; 1.53) | 0.72 | 0.95 |
|  |  | |  |  |  |  |  |
| Microbleeds | 0.81 (0.52 ; 1.26) | | 0.36 | 0.54 | 0.93 (0.61 ; 1.43) | 0.77 | 0.95 |
| Time | 1.05 (0.89 ; 1.25) | | 0.49 | 0.70 | 1.07 (0.89 ; 1.29) | 0.41 | 0.74 |
| Microbleeds*Time | 1.34 (0.91 ; 1.97) | | 0.13 | 0.46 | 1.26 (0.85 ; 1.88) | 0.24 | 0.66 |
|  |  | |  |  |  |  |  |
| Fractional anisotropy | 0.88 (0.73 ; 1.07) | | 0.21 | 0.47 | 0.97 (0.79 ; 1.19) | 0.80 | 0.95 |
| Time | 1.15 (0.98 ; 1.35) | | 0.07 | 0.46 | 1.11 (0.93 ; 1.32) | 0.21 | 0.66 |
| Fractional anisotropy*Time | 1.21 (1.03 ; 1.43) | | **0.01** | 0.27 | 1.14 (0.96 ; 1.36) | 0.12 | 0.66 |
|  |  | |  |  |  |  |  |
| Mean diffusivity | 1.23 (0.97 ; 1.56) | | 0.07 | 0.46 | 1.16 (0.87 ; 1.55) | 0.29 | 0.66 |
| Time | 1.16 (0.99 ; 1.35) | | 0.05 | 0.46 | 1.10 (0.92 ; 1.32) | 0.27 | 0.66 |
| Mean diffusivity*Time | 1.00 (0.85 ; 1.17) | | 0.99 | 0.99 | 1.01 (0.84 ; 1.22) | 0.83 | 0.95 |
|  |  | |  |  |  |  |  |

* False discovery rate adjusted p values

OR: odds ratio, CI: confidence interval, WMH: white matter hyperintensities

Model 1 was adjusted for age and sex. Model 2 adjusted for age, sex, education, smoking status, alcohol consumption, body mass index (BMI),

hypertension, type 2 diabetes, hyperlipidemia, heart diseases. Intracranial volume was also included in both two models for total brain volume,

gray matter volume, white matter volume, WMH, fractional anisotropy and mean diffusivity and Z scores were calculated for those variables.

The OR for the interaction term between a neuroimaging marker and time indicates the yearly increased chance of having clinically

relevant depressive symptoms with each standard deviation change in the respective neuro imaging marker.
